# Cushing’s syndrome caused by ACTH precursors secreted from a pancreatic yolk sac tumor in an adult—a case report and literature review

**DOI:** 10.3389/fmed.2023.1246796

**Published:** 2023-12-05

**Authors:** Johnny Yau Cheung Chang, Chariene Shao Lin Woo, Wing Sun Chow, Anne White, Ka Chung Wong, Po Tsui, Alan Chun Hong Lee, Eunice Ka Hong Leung, Yu Cho Woo, Kathryn Choon Beng Tan, Karen Siu Ling Lam, Chi Ho Lee, David Tak Wai Lui

**Affiliations:** ^1^Department of Medicine, Queen Mary Hospital, The University of Hong Kong, Pokfulam, Hong Kong SAR, China; ^2^School of Medical Sciences, Faculty of Biology, Medicine and Health, University of Manchester, Manchester, United Kingdom; ^3^Department of Pathology, Queen Mary Hospital, Pokfulam, Hong Kong SAR, China

**Keywords:** Cushing’s syndrome, ectopic ACTH syndrome, yolk sac tumor, pancreatic tumor, ACTH precursor

## Abstract

Here, we report the first adult case of pancreatic yolk sac tumor with ectopic adrenocorticotropic hormone (ACTH) syndrome. The patient was a 27-year-old woman presenting with abdominal distension, Cushingoid features, and hyperpigmentation. Endogenous Cushing’s syndrome was biochemically confirmed. The ACTH level was in the normal range, which raised the suspicion of ACTH precursor-dependent disease. Elevated ACTH precursors were detected, supporting the diagnosis of ectopic ACTH syndrome. Functional imaging followed by tissue sampling revealed a pancreatic yolk sac tumor. The final diagnosis was Cushing’s syndrome due to a yolk sac tumor. The patient received a steroidogenesis inhibitor and subsequent bilateral adrenalectomy for control of hypercortisolism. Her yolk sac tumor was treated with chemotherapy and targeted therapy. Cushing’s syndrome secondary to a yolk sac tumor is extremely rare. This case illustrated the utility of ACTH precursor measurement in confirming an ACTH-related pathology and distinguishing an ectopic from a pituitary source for Cushing’s syndrome.

## Introduction

Ectopic adrenocorticotrophic hormone (ACTH) syndrome, also termed paraneoplastic Cushing’s syndrome, can be caused by the secretion of ACTH and/or ACTH precursors from ectopic tumors. The tumors concerned secrete ACTH precursors, including unprocessed proopiomelanocortin (POMC) and POMC-derived peptides, owing to the altered post-translational processing of POMC (1). These tumors are associated with intense hypercortisolism and various complications, such as hypertension, hyperglycemia, osteoporosis, infection risks, and thrombotic tendencies (2). Distinguishing ectopic from pituitary-dependent Cushing’s syndrome is often challenging. The two conditions are classically distinguished by their variable responses to dynamic endocrine tests, including the high-dose dexamethasone suppression test, the corticotrophin-releasing-factor (CRF) test, and the desmopressin test (3). Pituitary imaging may sometimes provide a diagnosis if a pituitary macroadenoma is identified at this juncture. The gold standard for diagnosing pituitary Cushing’s is a positive inferior petrosal sinus sampling (IPSS) result. The measurement of ACTH precursors is reported to have diagnostic value in this scenario (4).

The most common source of ectopic ACTH is intrathoracic tumors, including bronchial carcinoid and small cell lung cancers. Other possible sources include gut neuroendocrine tumors and medullary thyroid cancer. Recognizing the potential causes of ectopic ACTH syndrome is essential as this provides guidance in locating the causative tumor and allows tumor-directed therapies. A yolk sac tumor as a cause of ectopic ACTH syndrome has only been reported in a 2-year-old child but not in adults (5). Here, we present a case of a 27-year-old Chinese woman who had Cushing’s syndrome due to ectopic ACTH precursor production from a pancreatic yolk sac tumor.

### Case description

**Figure 1:**
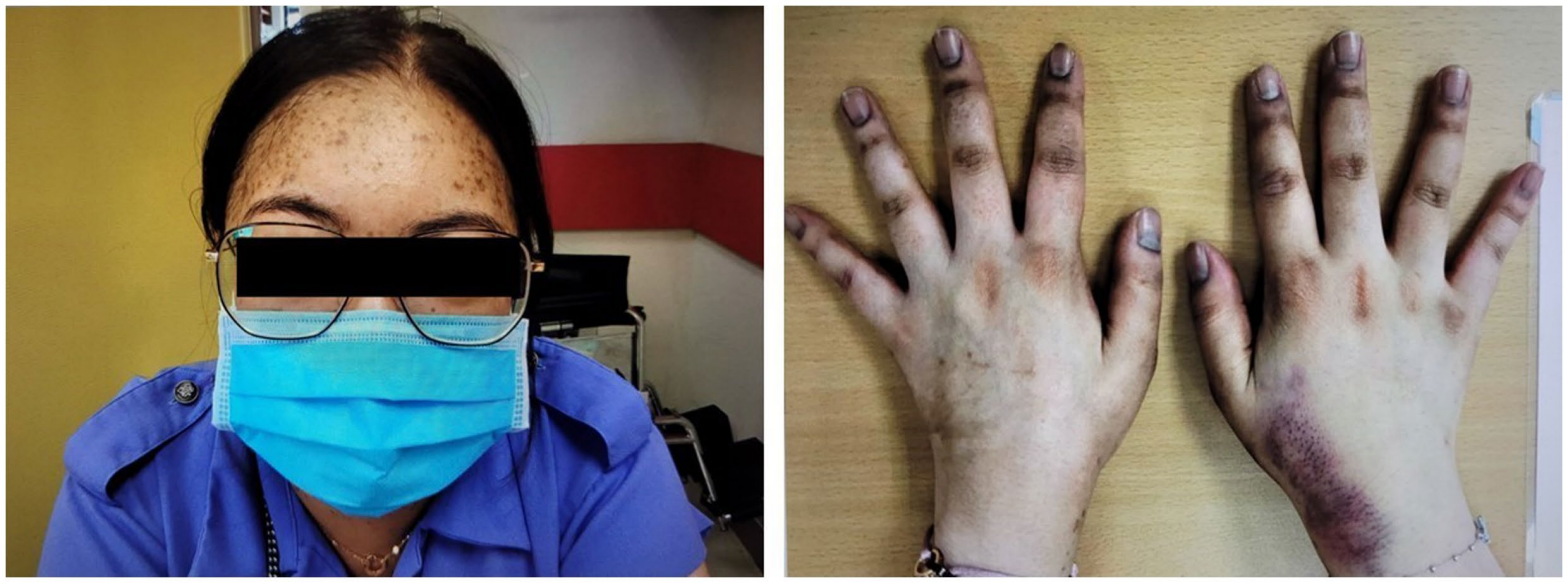
Cushingoid features at presentation include moon face, acne, thin skin, and easy bruising. Hyperpigmentation on the nails and knuckles was also noted.

A 27-year-old Chinese woman, who had unremarkable past health and family history, presented with right upper quadrant abdominal pain and nausea in early 2020. Abdominal ultrasonography was unrevealing. A few months later, she developed Cushingoid features and oligomenorrhea. At presentation, her blood pressure was 160/95 mmHg, body weight was 65.6 kg, and body mass index was 23.2 kg/m^2^. She had a moon face, hirsutism, proximal myopathy, bruising, thinning of the skin, and acne. She also had hyperpigmentation on the nails and knuckles of both hands (Figure 1).

### Diagnostic assessments

Her 9 am and 9 pm cortisol were both >1,700 nmol/L. Her 24-h urine-free cortisol was beyond the upper measurable limit at >1,500 nmol/L. Her serum cortisol was 759 nmol/L after a 1 mg overnight-dexamethasone suppression test, confirming endogenous Cushing’s syndrome. The morning ACTH was 35 pg/mL (upper limit of normal is 46 pg/mL). After excluding a high dose-hook effect, her blood sample was concomitantly sent for ACTH measurement using two different platforms to eliminate possible interference, which might cause a falsely low ACTH reading. ACTH was 19 pg/mL (upper limit of normal is 46 pg/mL) using an IMMULITE 2000 XPI, Siemens Healthineers, Erlangen, Germany, and 17 pg/mL (reference range: 7–63 pg/mL) using a Cobas e-801, Roche Diagnostics, Indianapolis, IN, United States, therefore verifying the ACTH measurement.

In view of this being ACTH-dependent Cushing’s syndrome, a high-dose-dexamethasone suppression test (HDDST) was performed, and her cortisol was not suppressed at 890 nmol/L, with ACTH 42 pg/mL. The serum cortisol day profile showed a mean cortisol level of >1,700 nmol/L (i.e., higher than the upper measurable limit of the assay) and an ACTH of 17 pg/mL. A CRF test using 100 μg of corticorelin showed less than a 50% rise in ACTH and no rise in cortisol levels (Supplementary Table S1). She suffered from multiple complications of hypercortisolism, including thoracic vertebral collapse with back pain, diabetes mellitus (HbA1c 6.7% and fasting glucose 7.6 mmol/L), and hypokalemic hypertension, with a lowest potassium level of 2.3 mmol/L.

The rapid onset of intense hypercortisolism and refractory hypokalemia, as well as the responses in the HDDST and CRF tests raised the suspicion of ectopic ACTH syndrome. Tumor markers were measured. Alpha-fetoprotein (AFP) was markedly raised at 33,357 ng/mL (reference range: <9 ng/mL). Beta-human chorionic gonadotropin (beta-hCG) was not elevated. Carcinoembryonic antigen (CEA) was 4.0 ng/mL (reference range: <3 ng/mL) and CA 19–9 was 57 U/mL (reference range: <37 U/mL). The marked hyperpigmentation in the context of normal ACTH levels pointed to the presence of an underlying tumor producing circulating ACTH precursors. Hence, magnetic resonance imaging (MRI) of the pituitary gland was not performed at this juncture. ACTH precursors were measured using a specialized immunoenzymatic assay (IEMA) employing in-house monoclonal antibodies against the ACTH region and the gamma MSH region. Both monoclonal antibodies have to bind to these regions in POMC and pro-ACTH to create a signal. The patient had a level of 4,855 pmol/L (upper limit of normal is 40 pmol/L) (6). This supported Cushing’s syndrome from an ectopic source secondary to an excess in ACTH precursors.

Localization studies were arranged to identify the source of ectopic ACTH precursors. Computed tomography (CT) of the thorax did not show any significant intrathoracic lesion but incidentally revealed a pancreatic mass. Dedicated CT of the abdomen confirmed the presence of a 7.9 × 5.6 cm lobulated mass in the pancreatic body; the adrenal glands were unremarkable. 18-FDG and 68Ga-DOTATATE dual-tracer positron-emission tomography-computed tomography (PET-CT) showed that the pancreatic mass was moderately FDG-avid and non-avid for DOTATATE (Supplementary Figure S1). Multiple FDG-avid nodal metastases were also present, including left supraclavicular fossa lymph nodes.

Fine needle aspiration of the left supraclavicular fossa lymph node yielded tumor cells featuring occasional conspicuous nucleoli, granular coarse chromatin, irregular nuclei, and a high nuclear-to-cytoplasmic ratio. Mitotic figures were infrequent. On immunostaining, the tumor cells were positive for cytokeratin 7 and negative for cytokeratin 20. Focal expression of CDX-2, chromogranin, and synaptophysin was noted. They were negative for TTF-1, GCDPF, Gata 3, Pax-8, CD56, ACTH, inhibin, and S-100 protein. Further immunostaining was performed in view of highly elevated AFP. The tumor cells expressed AFP, Sall4, and MNF-116. They were negative for c-kit, calretinin, Melan A and SF-1. Placental ALP (PLAP) was weak and equivocal. The features were in keeping with a yolk sac tumor.

### Therapeutic intervention and outcome

**Figure 2:**
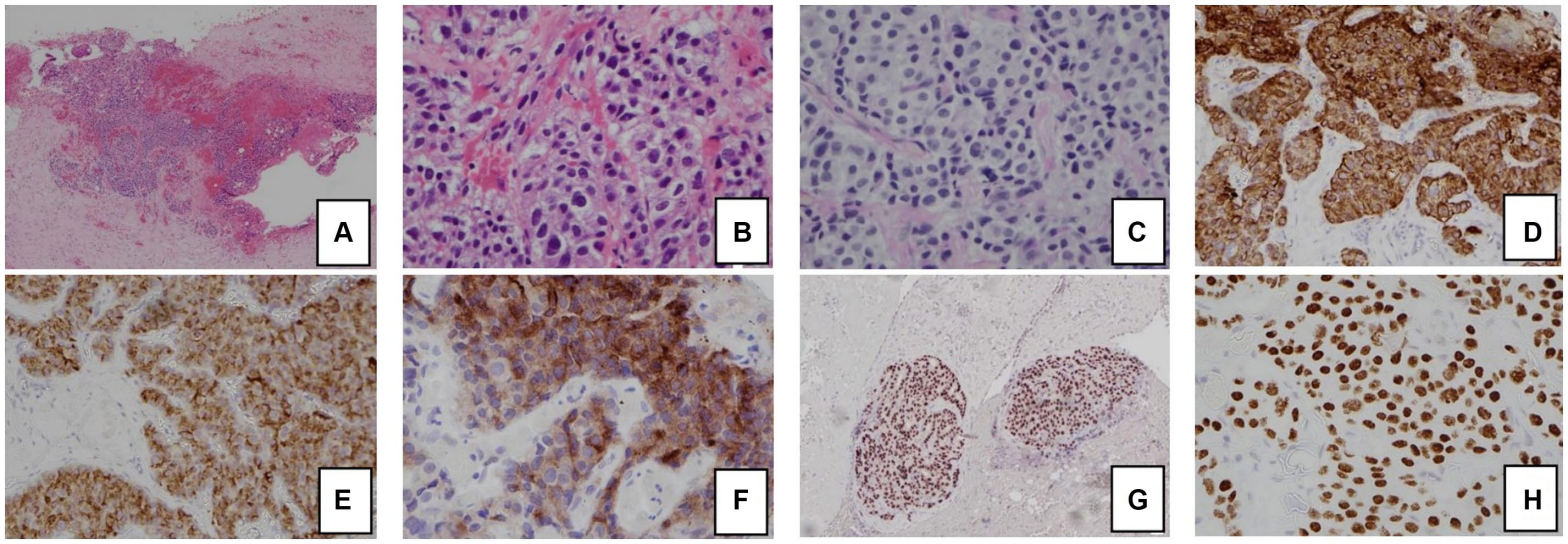
Histology and immunohistochemical staining pattern of tumor specimen. **(A)** HE stain x 40 showing tumor cells in the soft tissue and peritoneum. **(B)** HE × 400 showing that the tumor forms cords, nests, and ill-formed lumen in the vascular stroma. The tumor cells are polygonal with pale cytoplasm and pleomorphic nuclei. **(C)** PAS/D stain showing no cytoplasmic zymogen granules. **(D)** Tumor is diffusely positive for cytokeratin 7. **(E)** Tumor is positive for AFP. **(F)** Tumor is positive for glypican-3. **(G)** Tumor is diffusely positive for HNF1β. **(H)** Tumor is diffusely positive for SALL4.

The patient had significant hypokalemic hypertension requiring losartan 100 mg daily, spironolactone 100 mg daily, and a potassium supplement of 129 mmol/day. Co-trimoxazole was given for prophylaxis against *Pneumocystis jirovecii* pneumonia. Metyrapone was started and up-titrated to 1 gram three times per day. However, in view of persistent hypercortisolism, with urinary free cortisol persistently above the upper measurable limit of the assay, bilateral adrenalectomy was performed. The tumor was mainly in the periadrenal soft tissue, with vascular invasion. The tumor formed cords, nests, and ill-defined lumen (Figure 2). The tumor cells were polygonal and contained pale to eosinophilic cytoplasm and pleomorphic nuclei, some with large nucleoli. Mitosis was present while tumor necrosis was not obvious. The stroma was composed of vascular fibrous tissue, with minimal inflammatory reaction. Immunohistochemical study showed that the tumor was positive for cytokeratin 7, MNF-116, AFP, and glypican-3, and also positive for Sall4 and HNF1β. The tumor cells were negative for cytokeratin 20, PLAP, CD30, negative for neuroendocrine markers including S100 protein, synaptophysin, chromogranin, and also negative for Melan-A, inhibin, and ACTH. Histochemical study for Periodic acid–Schiff–diastase (PAS/D) showed no cytoplasmic zymogen granules like those of acinar cell tumor. The features were compatible with yolk sac tumor. She was put on glucocorticoid and mineralocorticoid replacements post-operatively.

**Figure 3:**
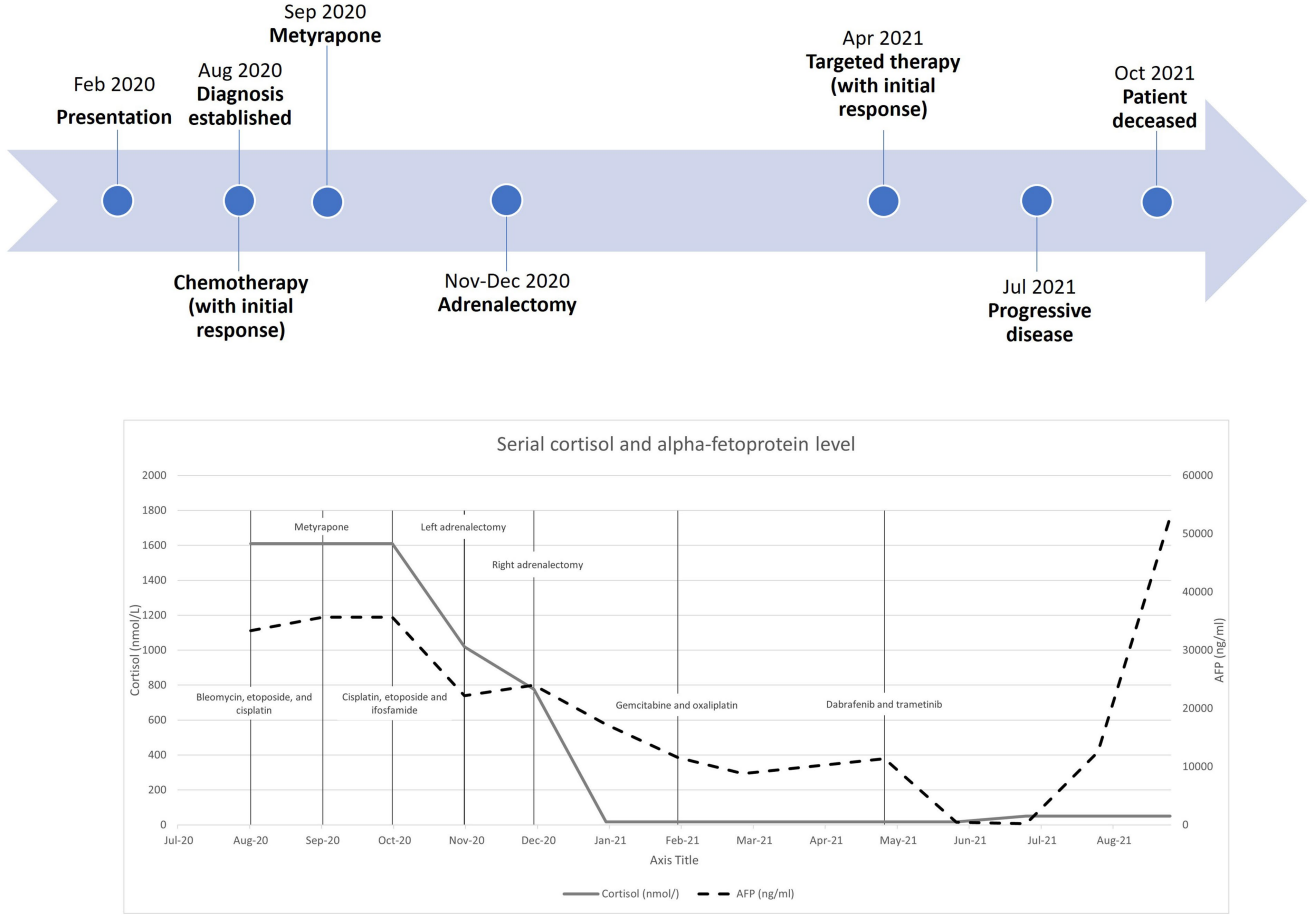
Timeline with serial cortisol and alpha-fetoprotein levels from diagnosis to patient death.

Regarding her oncological management, she received multiple lines of chemotherapy, but the response was poor. Due to limited access to the ACTH precursor assay, serial measurement was unavailable. Treatment response was monitored by repeated imaging and monitoring of AFP. Figure 3 shows a timeline indicating the key events of the disease, showing the trends of the AFP and cortisol levels. Apart from (i) bleomycin, etoposide, and platinum, she was sequentially treated with (ii) etoposide, ifosfamide with cisplatin, and (iii) palliative gemcitabine with oxaliplatin. Next-generation sequencing showed a BRAF V600E mutation, for which (iv) dabrafenib and trametinib were given. Unfortunately, the disease progressed, and the patient succumbed approximately one year after the disease was diagnosed.

## Discussion

This case demonstrates the diagnostic value of ACTH precursor measurement in the diagnosis of ectopic Cushing’s syndrome. ACTH precursors are raised in all ectopic tumors responsible for Cushing’s syndrome and could be useful in distinguishing ectopic from pituitary Cushing’s syndrome (4). Moreover, Cushing’s syndrome due to a yolk sac tumor has been reported only once in a pediatric case, and this is the first adult case reported in the literature (5).

POMC is sequentially cleaved in the anterior pituitary into pro-ACTH and then into ACTH, which is released into the circulation and binds to ACTH receptors in the adrenal cortex, leading to glucocorticoid synthesis (5, 7). Due to incomplete processing, ACTH precursors are found in normal subjects at a concentration of 5–40 pmol/L (6). Pituitary tumors are traditionally well-differentiated and can also relatively efficiently process ACTH precursors. However, this processing is less efficient in ectopic tumors that cause Cushing’s syndrome (8). Some less differentiated pituitary macroadenomas can secrete ACTH precursors into the circulation; however, these tumors are diagnosed by imaging and so do not, in general, cause problems with differential diagnosis (9).

Measurement of ACTH precursors by immunoradiometric assay (IRMA) was first described by Crosby et al. (10). The assay utilized monoclonal antibodies specific for ACTH and the other binding gamma-MSH. The assay only detects peptides expressing both epitopes and therefore measures POMC and pro-ACTH. The assay does not cross-react with other POMC-derived peptides such as beta-lipotropin, ACTH, and N-POMC.

**Figure 4:**
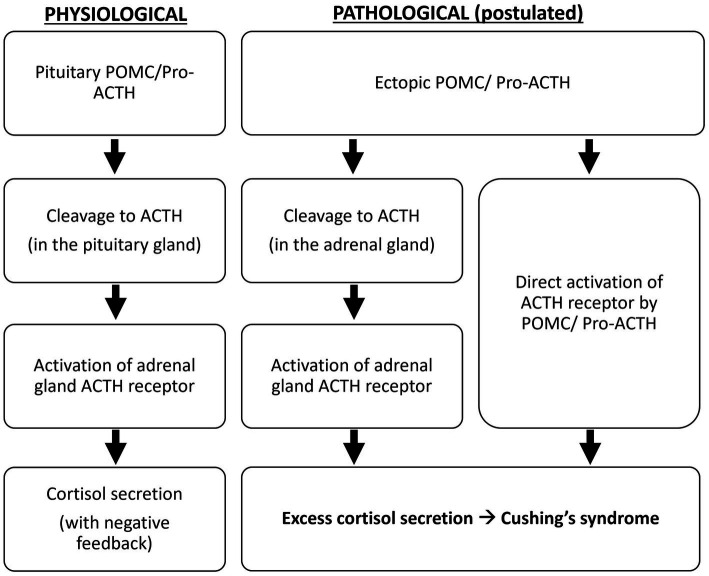
Postulated pathological mechanism of ectopic ACTH precursors.

Oliver et al. demonstrated that, compared to the pituitary adenomas in Cushing’s disease, all ectopic tumors responsible for Cushing’s syndrome in their study produce excessive POMC and pro-ACTH (4). The excessive production of ACTH precursors may reflect neoplasm-induced modification and amplification of POMC production. It is suggested that POMC binds to and activates the ACTH receptor because it contains the ACTH amino-acid sequence, or it is cleaved to ACTH in the adrenal glands to cause hypercortisolism (5) (Figure 4). Moreover, cleavage of POMC may produce peptides that exert mitogenic actions on adrenal cells and lead to adrenocortical growth. Outside the adrenal tissue, excessive ACTH precursors in Cushing’s syndrome caused by ectopic tumors can lead to marked hyperpigmentation. Both hypercortisolism and hyperpigmentation were observed in the reported case.

In patients with ACTH-dependent Cushing’s syndrome, ectopic tumors should be distinguished from pituitary tumors. The HDDST, at a cut-off of 50% cortisol suppression, gives a sensitivity of 81% and a specificity of 67% for pituitary dependent Cushing’s syndrome (11). The CRF test provides 82% sensitivity and 75% specificity for pituitary disease (8). IPSS is the gold standard in distinguishing pituitary from ectopic tumors in Cushing’s syndrome. Utilization of CRF-stimulated IPSS provides 93% sensitivity and 100% specificity for pituitary disease. It also allows correct lateralization in 78% of patients with pituitary tumors. However, it is only available in specialized centers.

In a retrospective cohort, the ACTH precursor level distinguished well between Cushing’s disease and ectopic ACTH syndrome (4). With a cut-off of 100 pmol/L, the test achieved 100% sensitivity and specificity for ectopic ACTH syndrome. More recently, this assay has been used to diagnose patients with occult ectopic ACTH syndrome, with ACTH precursors above 36 pmol/L (8). Unfortunately, the immunoassay for ACTH precursor measurement utilizes in-house monoclonal antibodies, which are not widely available.

Cross-reactivity of POMC in commercially available ACTH assays ranges from 1.6% to 4.7% (12). In cases of ectopic tumors causing Cushing’s syndrome with markedly raised ACTH-precursors and intense hypercortisolism, the cross-reactivity would give significantly high ‘ACTH’ measurements to suggest an ACTH-related pathology. The degree of cross-reactivity, which is variable, should ideally be provided by the assay manufacturer as it affects result interpretation. Lower levels of ACTH precursor production might not be detected, especially by assays with low precursor cross-reactivity. Clinical vigilance is crucial in reaching the correct diagnosis. In patients with marked hypercortisolism and a normal ACTH concentration, like in this case, the measurement of ACTH precursors would allow the accurate diagnosis of Cushing’s syndrome caused by ACTH precursors.

Ectopic tumors causing Cushing’s syndrome are associated with more intense hypercortisolism than Cushing’s disease (11). However, due to variable cross-reactivity, commercial ACTH assays might not accurately detect the excessive ACTH precursors responsible for the clinical syndrome. For this reason, ACTH measurements in these two conditions can significantly overlap and may not differentiate between ectopic and pituitary diseases (4). On the other hand, the more specific POMC assay described in 1996, which does not cross-react with pro-ACTH, has a low sensitivity of 80% for ectopic Cushing’s syndrome and is not now available (13). Hence, the ACTH precursor assay used in this reported case, which detects POMC and pro-ACTH, appears to provide the best diagnostic accuracy from the available literature.

Serial measurement of ACTH precursors may play a role in monitoring the treatment response in an ACTH precursor secreting tumor. In the case of ectopic ACTH secretion, the corticotropic axis is slowed down and ACTH is almost exclusively of paraneoplastic origin. Immunotherapy is known to alter the functioning of the hypothalamic–pituitary corticotropic axis; however, its effect on ectopic secretions is not known. More data is required before the role of ACTH precursor measurement for disease monitoring in these scenarios can be ascertained.

The incidence of endogenous Cushing’s syndrome is reported to be 2 to 4 per million people per year (14). Ectopic sources of Cushing’s syndrome are responsible for 9 to 18% of these cases. Typical sources of these ectopic tumors include bronchial carcinoid tumors, small-cell lung cancer, and gut neuroendocrine tumors. Notably, germ cell tumors, including teratomas, ovarian epithelial tumors, and ovarian endometrial tumors, are also possible ectopic sources of Cushing’s syndrome.

The histological diagnosis of germ cell tumor in a non-genital site is challenging, especially for the poorly differentiated, or with somatic differentiation. Immunostaining, chromosomal, or genetic study are very important in confirming the diagnosis. AFP elevation in our case limited the differential diagnoses to germ cell tumors/yolk sac tumors, hepatocellular carcinoma, and rare pancreatic tumors. The specimen was biopsied from the retroperitoneum, and the morphology was a dominant trabecular pattern or a hepatoid pattern. It showed diffuse positive immunostaining for cytokeratin, AFP, and glypican-3. It was also diffusely and strongly positive for HNF1β and SALL4, supporting the diagnosis of yolk sac tumor. Both HNF1β and SALL4, being related with the expression of genes associated with stem cells or progenitor cells, are used as sensitive and specific markers for germ cell tumors/yolk sac tumors (15, 16).

Staining related to pancreatic acinar cell carcinoma and neuroendocrine tumor were performed. PAS/D staining showed a lack of zymogen granules. A lack of nuclear β-catenin positivity was shown. Staining for neuroendocrine markers, including chromogranin and synaptophysin, was negative. Bcl-10 and trypsin were not available in the local setting.

Cushing’s syndrome due to a yolk sac tumor was reported only once, in a 2-year-old child (5). The abdominal yolk sac tumor was resistant to cisplatin, with rapid disease progression, and the patient succumbed 1.5 years after initial presentation. Yolk sac tumor in the pancreas is also rare, with only 4 cases reported so far. The first case was reported in a 57-year-old woman with an incidentally detected abdominal mass (17). The tumor stained positive for AFP, PLAP, and CEA. The second case was a 70-year-old asymptomatic woman with histology showing a group of tumor cells with features of a yolk sac tumor, and another group showing features of pancreatic ductal adenocarcinoma with mucin production, suggesting a yolk sac tumor derived from pancreatic ductal adenocarcinoma (18). The tumor showed partial positivity for AFP, Sall4, glypican-3, and cytokeratin 7, as found in our case, while MNF-116 and PLAP staining results were not described. The third was in a 33-year-old man with a solitary pancreatic head mass with obstructive jaundice (19). The patient had undergone Whipple’s procedure followed by cisplatin-based chemotherapy, resulting in at least 5 years of disease remission. The latest reported case was in a 32-year-old man presenting with abdominal pain (20). Notably, initial imaging showed diffuse enlargement of the pancreas and increased FDG uptake without a distinct mass. Reassessment imaging 11 months later showed a 13 cm pancreatic mass. The initial imaging findings suggested initial intraductal growth of the tumor, as reported in some subtypes of pancreatic carcinoma. None of the reported cases of adult pancreatic yolk sac tumors were associated with abnormal hormone secretion. We reported the first adult case of pancreatic yolk sac tumor with ectopic ACTH syndrome. The case represents an overlap of two rarities. It demonstrates that pancreatic yolk sac tumor is a possible cause of ectopic ACTH syndrome.

## Conclusion

ACTH precursor measurement helps to distinguish ectopic ACTH syndrome from Cushing’s disease. The test has superior diagnostic performance and is less invasive than IPSS. Nonetheless, the limited availability of the assay may restrict its broader use in patient management. We describe the first adult case of pancreatic yolk sac tumor with ACTH precursor secretion resulting in Cushing’s syndrome. This adds to the list of origins of ectopic ACTH syndrome in adults.

## Data availability statement

The original contributions presented in the study are included in the article/Supplementary material, further inquiries can be directed to the corresponding author.

## Ethics statement

Written informed consent was obtained from the individual to publish any potentially identifiable images or data in this article.

## Author contributions

JC wrote the manuscript. JC, CW, WC, AW, KW, and PT researched the data. WC, AL, EL, YW, KT, KL, and CL critically reviewed and edited the manuscript. DL initiated and conceptualized this case report and is the guarantor of this work. All authors contributed to the article and approved the submitted version.
