# Supplementary material for: Cushing’s syndrome caused by ACTH precursors secreted from a pancreatic yolk sac tumor in an adult—a case report and literature review
Source: Front Med (Lausanne). 2023 Dec 5;10:1246796. doi: 10.3389/fmed.2023.1246796 (PMC10728467; doi:10.3389/fmed.2023.1246796)
Supplement: Supplementary file 1 [file Table_1.DOCX]

**Table 1**. Corticotropin-releasing factor test

| **Time (minutes)** | -15 | 0 | 15 | 30 | 45 | 60 | 90 | 120 |
| --- | --- | --- | --- | --- | --- | --- | --- | --- |
| **ACTH (pg/mL)** | 23 | 24 | 20 | 21 | 19 | 19 | 19 | 19 |
| **Cortisol (nmol/L)** | 5050 | 4730 | 4620 | 4530 | 4460 | 4500 | 4270 | 4200 |
