# Supplementary figures and images for: Cushing’s syndrome caused by ACTH precursors secreted from a pancreatic yolk sac tumor in an adult—a case report and literature review
Source: Front Med (Lausanne). 2023 Dec 5;10:1246796. doi: 10.3389/fmed.2023.1246796 (PMC10728467; doi:10.3389/fmed.2023.1246796)

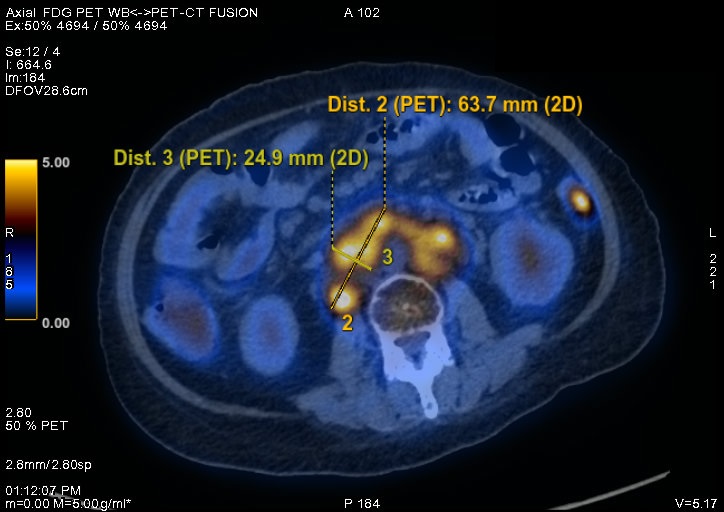

Supplement: Supplementary file 2 [file Image_1.JPEG]
